# T‐cell non‐Hodgkin’s lymphoma of both breasts: An uncommon presentation of a common disease

**DOI:** 10.1002/ccr3.4048

**Published:** 2021-03-13

**Authors:** Senai Goitom Sereke, Anthony Oriekot, Vincent Mboizi, Naghib Bogere, Felix Bongomin

**Affiliations:** ^1^ Department of Radiology and Radiotherapy School of Medicine Makerere University College of Health Sciences Kampala Uganda; ^2^ Uganda Cancer Institute Kampala Uganda; ^3^ Department of Medicine School of Medicine Makerere University College of Health Sciences Kampala Uganda; ^4^ Department of Medical Microbiology and Immunology Faculty of Medicine Gulu University Gulu Uganda

**Keywords:** both breasts, breast, lymphoma, metastatic

## Abstract

Secondary T‐cell non‐Hodgkin's lymphoma of the breasts is a very rare disease and can be easily missed as inflammatory carcinoma at initial presentation. High index of suspicion and radiological investigations has a big role in identifying the primary lymphoma.

## Graphical abstract

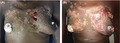

## 1 INTRODUCTION

An 18‐year‐old girl presented with hypopigmentation, ulcerative skin changes, and masses of both breasts for over a year. She was referred to the Uganda Cancer Institute (UCI) with suspected inflammatory breast cancer. Radiological findings and Tru‐cut biopsy were consistent with secondary CD3 + CD20‐ T‐cell NHL of both breasts.

Primary or secondary lymphoma of the breast is rare and is usually of non‐Hodgkin's lymphoma (NHL) subtype.^1^ Secondary lymphoma of the breast that arises elsewhere in the body is very rare and the reported incidence is about 0.07%.^2^ In the literature, less is known about secondary lymphoma than primary lymphoma of the breast.^3^ Lymphoma of the breast usually presents as a painless mass. However, T‐cell lymphoma could mimic the clinical presentation of inflammatory breast carcinoma (*mastitis carcinomatosis*) or mastitis and abscess of infectious etiologies.^4^

Triple assessment is key for all women presenting with breast masses or suspicious lesions. Breast ultrasound is cheap and rapid allowing early identification of small lesions and guides biopsy sampling for histology—the confirmatory test.^5^ The majority of the cases of breast lymphomas are unilateral; however, secondary and Burkitt's lymphoma tends to be bilateral.^6^ Herein, we present a case of secondary NHL of both breasts diagnosed in a teenage Ugandan woman.

## 2 CASE PRESENTATION

An 18‐year‐old girl from eastern Uganda was referred to the Uganda Cancer Institute (UCI) with complaints of hypopigmentation of the skin of both of her breasts, which was followed by ulceration and visible masses for 14 months. The masses were painless for the first eight months but became so painful in the last 6 months prior to her presentation. She had been treated as a case of a possible bacterial mastitis with topical and oral antibiotics for two months without any significant improvement and was referred to UCI with a clinical suspect for bilateral inflammatory breast cancer. However, due to financial challenges the patient did not reach UCI until a year later. By then, she had developed a serosanguinous nipple discharge, generalized body weakness, weight loss, and lower limb swelling extending up to the knees. She had no cough, chest pain, night sweats, or fever. She had no prior history of chronic illness or a family history of a cancer. Her menarche was at the age of 14 years.

**FIGURE 1:**
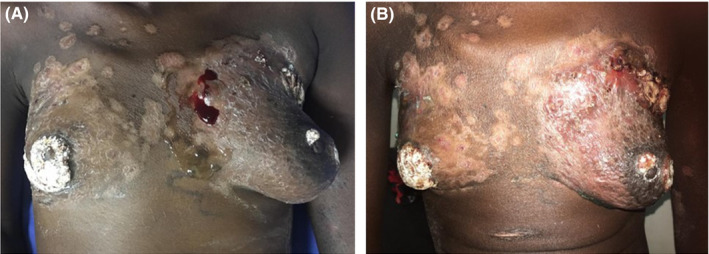
A and B demonstrated hypopigmentation of skin in nonuniform pattern with dried whitish powder covering both nipples. The bleed in the left upper inner quadrant during echocardiography

On physical examination, the patient was cachectic and mildly pale with normal vital signs. She had multiple, firm matted lymph nodes in the right posterior triangle of the neck, both axillae and bilateral inguinal regions. There were no supraclavicular enlarged lymph nodes. The breasts had hypopigmentation with visible bleeding in some areas. The nipples and areolae of both breasts were coated with layers of a whitish powder (Figure 1). There were multiple small masses in both breasts that were irregular, tender, and firm‐to‐hard in consistency. Chest examinations were essentially normal except for a right basal crepitation. Both lower extremities demonstrated pitting edema up to the knee.

Complete blood count showed hemoglobin of 10.9 g/dL, total white cell count of 13 680/dL, and platelet count of 460 000/dL. Blood chemistry showed blood urea nitrogen, 263 mg/dL; creatinine, 1 mg/dL; lactate dehydrogenase (LDH), 2010 U/L; and serum alkaline phosphatase, 173 U/L.

**FIGURE 2:**
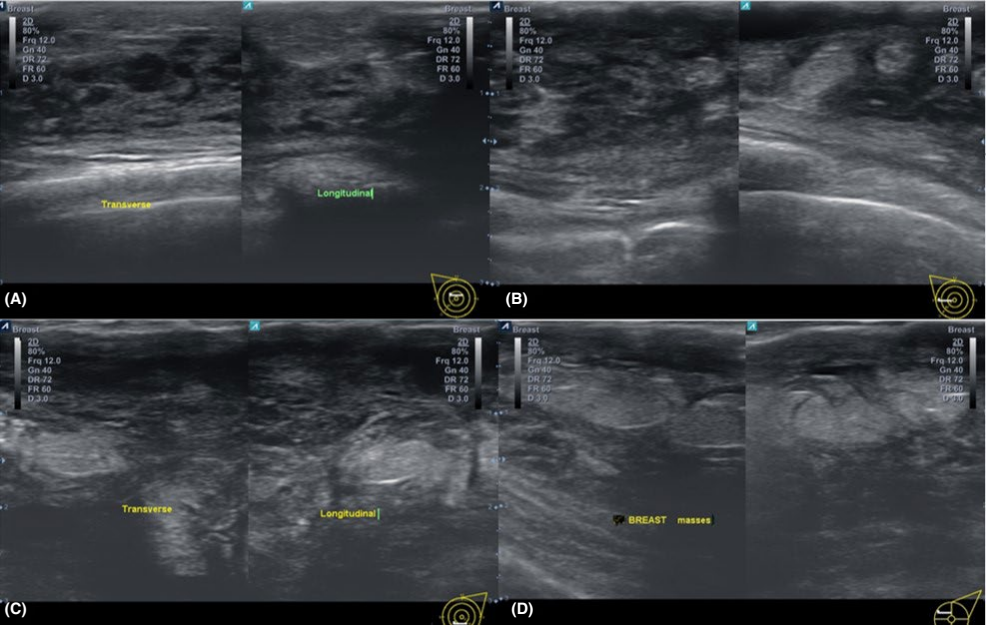
A, B, C, and D ultrasound of both breasts. A and B, Breast ultrasound of the right breast demonstrated multiple ill‐defined more hypoechoic lesions and less hyperechoic lesions at 12 O’clock above the nipple and at 9 O’clock, respectively. There were also subcutaneous lesions, skin thickening, and posterior enhancement. C and D—Breast ultrasound of the left breast demonstrated multiple ill‐defined more hyperechoic lesions and less hypoechoic lesions at 6 O’clock below the nipple and at 10 O’clock, respectively. There were subcutaneous involvement and skin thickening which are lesser than that of the right breast

Breast ultrasound examination demonstrated bilateral tender breasts with multiple hyperechoic and hypoechoic lesions of ill‐defined margins, subcutaneous lesions, and skin thickening in both breasts. There were multiple matted enlarged hypoechoic lymph nodes with very small hilum in some of the nodes in both axillae (Figure 2).

**FIGURE 3:**
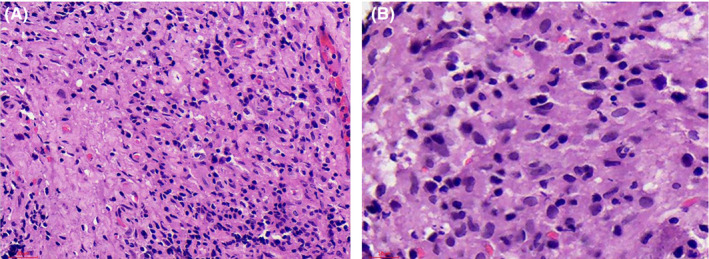
Hematoxylin and eosin A, 40× magnification, B, 80× magnification. A, Shows tumor with foamy histiocytes in the background and lymphoid cells. B, Shows tumor with lymphoid cells with moderate pleomorphism. No Reed Sternberg cells were seen

Since inflammatory breast cancer was suspected, an ultrasound‐guided Tru‐cut biopsy of the lumps in both breasts was performed. Hematoxylin and eosin section (Figure 3) showed effacement of the normal architecture by a diffuse infiltrate of large, pleomorphic lymphoid cells with vesicular chromatin and prominent nucleoli, and no Reed Sternberg cells were seen. Immunohistochemistry showed cluster of differentiation (CD)‐3 positivity and CD20 negative.

Ultrasound scan of the abdomen for staging showed hepatosplenomegaly, mesenteric, para‐aortic and bilateral inguinal lymphadenopathy, and a well‐defined, solid, hypoechoic pelvic mass originating from the fundus of the uterus with no color flow on Doppler. The left kidney was echogenic with dilated calyces but of normal size.

Chest radiographs demonstrated right middle and lower lung zone reticulo‐nodular opacities with thickened horizontal fissure. The retrosternal space was filled with homogenous opacity.

Echocardiography demonstrated normal cardiac structure and function. However, multiple matted lymph nodes in the anterior and superior mediastinum were seen during echocardiography examination.

The histopathologic and radiographic investigations confirmed a stage IV T‐cell NHL with secondary extranodal involvement of both breasts. However, her family unanimously declined medical management and decided to take her back home.

## 3 DISCUSSION

Here, we reported a case of a secondary T‐cell NHL of both breasts—a rare presentation of NHL and a diagnostic chameleon of a primary breast cancer. To help clinicians to recognize similar case in their clinical practice, we discuss the presentation, investigation, and treatment options of breast lymphoma.

Breast lymphomas can be primary or secondary. They rarely affect the breasts, but when they do, they are usually secondary.^3^ The commonest histological subtype is diffuse large B‐cell type (DLBCL), and the age of incidence is between 9 and 85 years with median age range between 55 and 65 years.^4^, ^7^ Our patient was 18 years old which is in the age range but with huge gap from median age. There was a delay of over a year in the establishment of the final diagnosis.

Primary breast lymphoma is diagnosed if there is only involvement of one breast with or without ipsilateral axillary involvement; however, bilateral breast involvement suggests secondary involvement.^8^, ^9^ Our patient presented with systemic lymphatic involvement mainly in the neck, anterior mediastinum, mesenteric and para‐aortic, and inguinal involvement. Moreover, there was hepatosplenomegaly with echogenic left kidney which was suggestive of systemic lymphoma.

Painless palpable mass is the commonest symptom of breast lymphoma with rare presentation of nipple retraction or discharge and skin change.^10^ T‐cell lymphomas are more commonly associated with skin changes, edema, and localized tenderness than the B‐cell lymphomas of the breast.^4^ The patient presented with significant skin changes with palpable masses and nipple discharge bilaterally. The masses were painful even if her vest touched the area.

Breast imaging, especially breast ultrasound characteristics, may aid in the diagnosis of breast lymphoma.^4^ Ultrasound can demonstrate a hypoechoic solid mass with circumscribed or indistinct margins. Bilateral axillary lymphadenopathy or breast edema on ultrasound could indicate a secondary lymphoma. There are no specific mammographic features for breast lymphoma.^4^, ^11^, ^12^

It is not easy to distinguish breast lymphoma from various benign or malignant breast diseases on the basis of clinical and radiological findings especially if there is no known systemic lymphoma and patients present with a painful breast lump, erythema, or skin thickening.^8^ Our patient was initially suspected to have bilateral inflammatory breast carcinoma by her primary physician.

The management of lymphoma of the breast is controversial.^13^ Secondary lymphoma of the breast is commonly associated with multiorgan involvement.^14^ Treatment options include mainly combination chemotherapy with or without radiotherapy or at times radiotherapy alone.^15^

## 4 CONCLUSION

Secondary T‐cell NHL of the breasts is a very rare disease and can be easily missed as inflammatory carcinoma of the breast at initial stage of the disease. High index of suspicion and radiological investigations have a big role in identifying the primary systemic lymphoma, as there are no pathognomonic radiologic features.

## CONFLICT OF INTEREST

The authors declare that they have no competing interests.

## AUTHOR CONTRIBUTIONS

SG, AO, VM, NB, and FB: made substantial contributions to conception and design, acquisition of data, or analysis and interpretation of data; took part in drafting the article or revising it critically for important intellectual content; agreed to submit to the current journal; gave final approval of the version to be published; and agree to be accountable for all aspects of the work.

## CONSENT FOR PUBLICATION

We obtained an informed consent from the patient herself and the mother of the patient to publish this case report.

## Data Availability

The information used and/or analyzed during this case report is available from the corresponding author on reasonable request.
